# Evaluation of a novel optical smartphone blood pressure application: a method comparison study against invasive arterial blood pressure monitoring in intensive care unit patients

**DOI:** 10.1186/s12871-022-01797-0

**Published:** 2022-08-15

**Authors:** Olivier Desebbe, Chbabou Anas, Brenton Alexander, Karim Kouz, Jean-Francois Knebel, Patrick Schoettker, Jacques Creteur, Jean-Louis Vincent, Alexandre Joosten

**Affiliations:** 1 Department of Anesthesiology and Intensive Care, Clinique de La Sauvegarde, 80 Avenue Ben Gourion, 69009 Lyon, France; 2 Department of Anesthesiology, CUB Erasme University Hospital, Université Libre de Bruxelles, 808 Route de Lennik, 1070 Brussels, Belgium; 3 Department of Anesthesiology and Perioperative Care, University of California San Diego, San Diego, CA USA; 4 Department of Anesthesiology, Center of Anesthesiology and Intensive Care Medicine, University Medical Center Hamburg-Eppendorf, Martinistrasse 52, 20246 Hamburg, Germany; 5 Biospectal SA, 1003 Lausanne, Switzerland; 6 Department of Anesthesiology, Lausanne University Hospital and University of Lausanne, Lausanne, Switzerland; 7 Department of Intensive Care, CUB Erasme University Hospital, Université Libre de Bruxelles, 808 Route de Lennik, 1070 Brussels, Belgium; 8 Department of Anesthesiology, Paul Brousse Hospital, Université Paris-Sud, Villejuif, France

**Keywords:** Arterial hypertension, mobile phone, Mobile health, Hemodynamic, Hemodynamic monitoring, Optical signal, International standards

## Abstract

**Background:**

Arterial hypertension is a worldwide public health problem. While it is currently diagnosed and monitored non-invasively using the oscillometric method, having the ability to measure blood pressure (BP) using a smartphone application could provide more widespread access to hypertension screening and monitoring. In this observational study in intensive care unit patients, we compared blood pressure values obtained using a new optical smartphone application (OptiBP™; test method) with arterial BP values obtained using a radial artery catheter (reference method) in order to help validate the technology.

**Methods:**

We simultaneously measured three BP values every hour for five consecutive hours on two consecutive days using both the smartphone and arterial methods. Bland–Altman and error grid analyses were used for agreement analysis between both approaches. The performance of the smartphone application was investigated using the Association for the Advancement of Medical Instrumentation (AAMI) and the International Organization for Standardization (ISO) definitions, which require the bias ± SD between two technologies to be below 5 ± 8 mmHg.

**Results:**

Among the 30 recruited patients, 22 patients had adequate OptiBP™ values and were thus analyzed. In the other 8 patients, no BP could be measured due to inadequate signals. The Bland–Altman analysis revealed a mean of the differences ± SD between both methods of 0.9 ± 7 mmHg for mean arterial pressure (MAP), 0.2 ± 14 mmHg for systolic arterial pressure (SAP), and 1.1 ± 6 mmHg for diastolic arterial pressure (DAP). Error grid analysis demonstrated that the proportions of measurement pairs in risk zones A to E were 88.8% (no risk), 10% (low risk), 1% (moderate risk), 0% (significant risk), and 0% (dangerous risk) for MAP and 88.4%, 8.6%, 3%, 0%, 0%, respectively, for SAP.

**Conclusions:**

This method comparison study revealed good agreement between BP values obtained using the OptiBP™ and those done invasively. The OptiBP™ fulfills the AAMI/ISO universal standards for MAP and DAP (but not SAP). Error grid showed that the most measurements (≥ 97%) were in risk zones A and B.

**Trial registration:**

ClinicalTrials.gov registration: NCT04728477

## Background

Arterial hypertension is a leading predicting factor of poor health outcome worldwide, in both developed and developing countries [1–3]. In 2020, just over 1.4 billion adults had hypertension, but it is estimated that only 46% of people with arterial hypertension are aware of having the disease [4]. Arterial hypertension and its complications (e.g., kidney failure, stroke and heart failure) are responsible for significant resource use across healthcare systems worldwide. Arterial hypertension is most commonly diagnosed using automatic ambulatory non-invasive blood pressure (BP) measurements, with oscillometry and a pressure cuff around the arm or wrist being the most frequently used method. Healthcare systems in most developed countries encourage self-measurement of BP at home for a wide variety of reasons, such as enabling more frequent measurements, eliminating the white coat effect, detecting masked arterial hypertension, and facilitating better titration of treatment changes by the physician, which may ultimately reduce cardiovascular complications [5–7].

The technological capabilities of our “smartphones” have evolved dramatically in recent years, which has led to the emergence of multiple health applications that can be used in the diagnosis, prevention, and management of several diseases [8, 9]. Newer mobile phone applications are now able to numerous vital signs non-invasively [10, 11] and even display flow variables or dynamic parameters of fluid responsiveness [12–15]. As such, having such smartphone application widely available may one day improve the management of arterial hypertension without the current bulky and costly devices. Considering that at least 6.6 billion people own a smartphone worldwide, an accurate and reliable method to measure BP using a smartphone would enable a large percentage of the world's population to have easy access to arterial hypertension screening and monitoring.

A new optical smartphone application for BP measurement (OptiBP™) has recently been developed by Biospectal (Lausanne, Switzerland) and tested against upper arm cuff oscillometry [16–19], but no studies have compared OptiBP™-derived BP values with invasive BP values obtained using an arterial catheter as the reference method. We therefore conducted an observational study in intensive care unit (ICU) patients to compare BP values obtained with the OptiBP™ with those obtained invasively using an arterial catheter.

## Methods

This prospective study was registered on ClinicalTrials.gov on January 28^th^, 2020 under the reference NCT04728477 (Principal Investigator: Alexandre Joosten).The Erasme Ethics Committee approved the study on February 2, 2021 under the reference A2020/665. The study took place between February 3 and April 1, 2021. We had written informed consent from each patient or their next of kin if a patient was unable to do it.

We included all adult patients having invasive arterial BP monitoring using a radial artery catheter for at least 48 h. Exclusion criteria were patients with an inter-arm BP difference > 10 mmHg in systolic arterial pressure (SAP) measures using a brachial cuff, patient with dementia, psychological disorders, drug or alcohol abuse unless receiving mechanically ventilation, patient with atrial fibrillation and patient with finger lesions that would alter the correct capture of signals by the mobile phone.

### OptiBP™

**Fig. 1:**
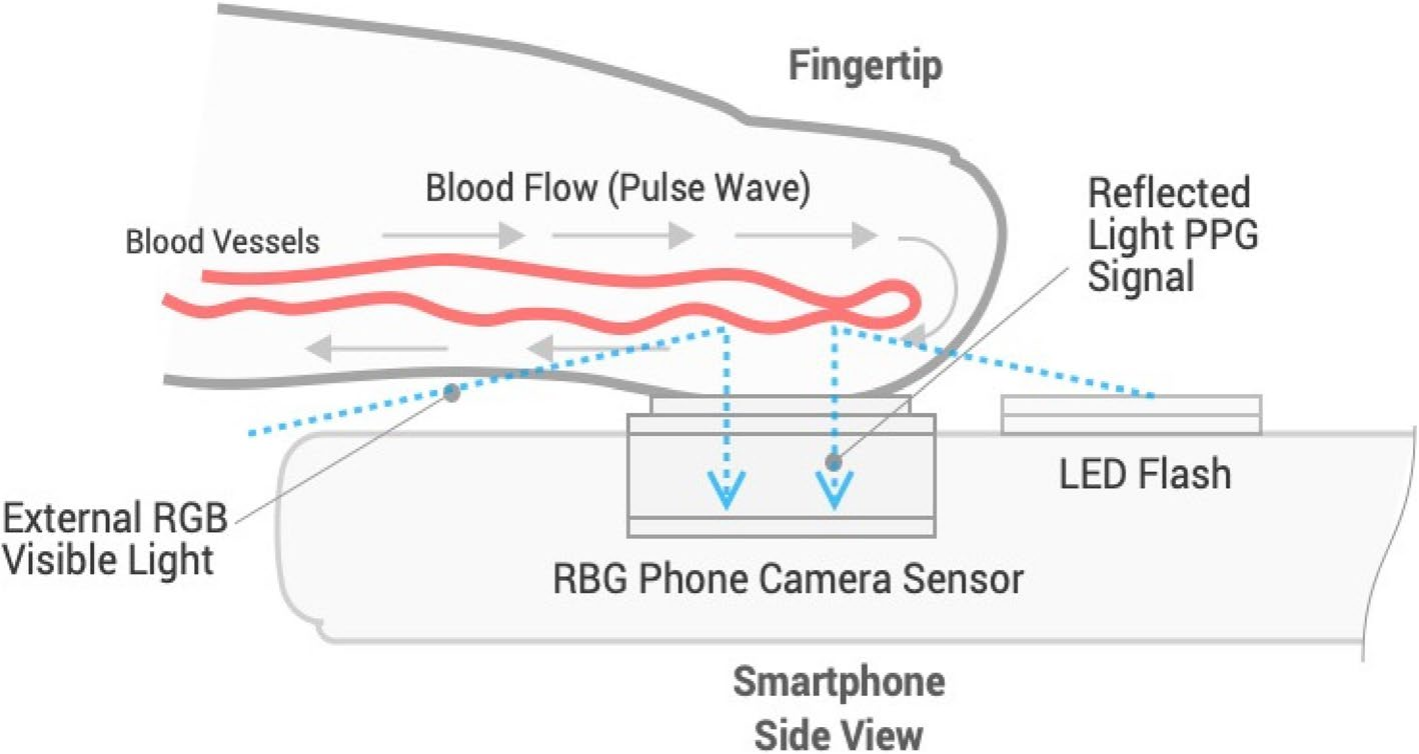
Description of the smartphone application: Fingertip on the smartphone’s camera OptiBP™ app uses image data generated from volumetric blood flow changes via light passing through the fingertip, reflecting off blood flowing through the vessels, and then passing to the phone camera's image sensor

OptiBP™ is the name of the smartphone application used in the current study and it is an acronym of "optical blood pressure" (Biospectal Inc., Lausanne, Switzerland). This software was deployed on a Samsung Galaxy S7 smartphone (Samsung GEC, Samsung Seocho Town, Seocho-gu, Seoul, Korea). Previous validation studies have been completed that describe how this technology estimates BP from pulse wave analysis of pulse oximetry signals [20]. An algorithm (CSEMBP: optical BP monitoring) analyses smartphone-derived photo-plethysmography (PPG) signals generated by the light from the smartphone’s camera flash that enters the finger, is refracted by the tissue, and is finally recorded by the smartphone camera. A brief description of the technology will be provided, but more information can be found in our prior studies: The OptiBP™ application records high-speed video sequences of PPG signal changes that are generated from volumetric changes in blood flow in the finger (Fig. 1). Each pulse of a 30-s PPG signal is assigned a quality index and then averaged, thus obtaining pulse wave estimates with the highest possible quality for each period. Subsequently, each accepted pulse wave passes through a bank of time-derived filters, allowing characterization of morphological variations in the pulse at different temporal resolutions. The algorithm can provide absolute changes in BP relative to an arbitrary baseline value but requires an initial calibration procedure (using a validated BP collection method) to define this baseline value and to obtain further absolute BP values.

### Invasive arterial lines

The Dräger Infinity Delta XL (Wemmel, Belgium) was used to monitor patients during their ICU stay. The invasive arterial BP signal (reference BP value) was obtained using a radial artery catheter.

### Study protocol

Patients were managed according to standard practice throughout the study period. Before starting the study, the pressure transducer was zeroed or leveled and the dynamic response of the system was checked. When the patient was calm and not agitated, three BP values were simultaneously recorded every hour over a five-hour period using the two methods. This process was repeated the following day (giving 10 time points in total). The duration of each measurement was 30 s with one-minute break in between. The first three measurements were used as calibration with the following nine measurements used for analysis. At least one OptiBP™ value needed to be usable (with correct values) at each time point in order for a patient to be included in the final analysis. The smartphone technology was used on the opposite arm to that of the radial artery catheter.

### Statistical analysis

No sample size was calculated for this study. However, the European Society of Hypertension [21] recommends a minimum of 20 patients for a study such as ours. Incorporating potential dropout, we chose to recruit 30 patients here.

Patient characteristics are presented as mean ± standard deviation (SD) or absolute number and percentage (%). SAP, diastolic arterial pressure (DAP), and mean arterial pressure (MAP) values obtained with the OptiBP™ were compared with invasive arterial measurements with Bland–Altman analysis by calculating the bias (BP of the test method minus BP of the reference method) together with SD, and 95% limits of agreement (mean of the difference ± 1.96 × SD) accounting for repeated measurements. We assessed the performance of the OptiBP™ with the ISO standards, which require the bias between the test and the reference method to be less ≤ 5.0 mmHg ± 8.0 mmHg [21].

Error grid analysis recently proposed by Saugel et al. [22] was done on data. This analysis consists of a scatterplot with reference BP measurements on the x-axis and measurements from the test method on the y-axis overlaid on a grid that is divided into five risk zones (zones A to E). Each BP measurement pair was categorized into one of the five risk zones, which describes the potential clinical risk caused by a difference in the BP measured using the test method and that measured using the reference method. These five zones are color-coded from green (zone A, no risk) to red (zone E, life-threatening risk).

All statistics were performed using Excel and MedCalc® Statistical Software version 19.6.4 (MedCalc Software Ltd, Ostend, Belgium), and the error grid analysis was done using Matlab (The MathWorks Inc, Natick, MA, USA).

## Results

Thirty patients were enrolled in the study. Among them, the OptiBP^TM^ was able to capture at least one reliable BP value per measurement time point in 22 patients (73%) which were used for statistical analysis (8 patients (27% of the study collective) had inadequate OptiBP^TM^ signals). Patient characteristics are shown in Table 1.

**Table 1:** Patients’ baseline characteristics

Patients’ characteristics	(*N* = 22)
Age (years)	61 [53-65]
Sex, Male	7 (32%)
Height (cm)	171 ± 9
Body weight (kg)	78 ± 17
Body mass index (kg/m^2^)	32 [27–37]
Hypertensive patients	14 (23%)
Treated for hypertension	14 (23%)
Hyperlipidemia	12 (54%)
Diabete II	8 (36%)
Chronic renal insufficiency	4 (18%)
Patients under noradrenaline	7 (33%)
Reason for ICU admission	
- COVID-19 infection	14 (63%)
- Postoperative period of a neurosurgical procedure	3 (13%)
- Postoperative period of a cardiac surgery	2 (9%)
- Multi-organ failure	1 (4%)
- Acute respiratory insufficiency	1 (4%)
- Postoperative period of a liver transplant surgery	1 (4%)

Variables are presented as mean ± *SD*, median [25–75] percentiles or absolute number (percentage)

**Fig. 2:**
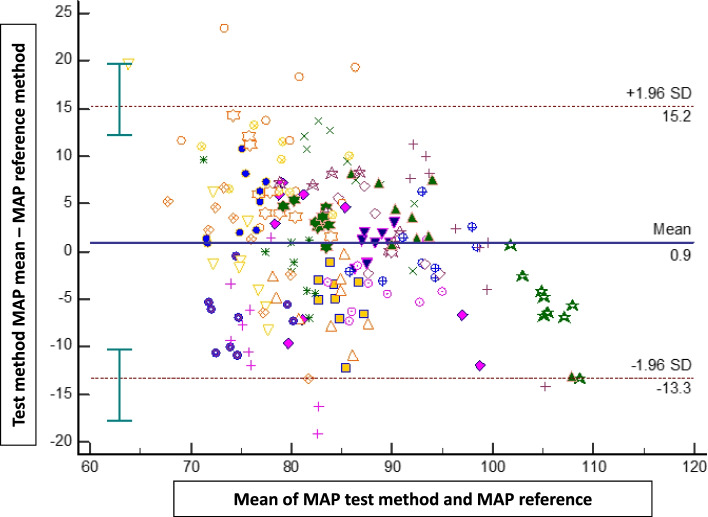
Bland–Altman plots showing the agreement between the smartphone application for mean arterial pressure (MAP) measurements and the reference method (upper arm oscillometry)

**Fig. 3:**
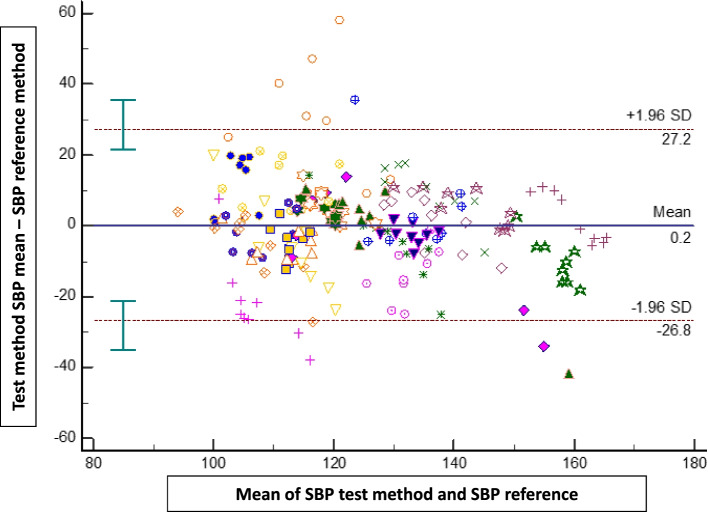
Bland–Altman plots showing the agreement between the smartphone application for systolic blood pressure (SAP) measurements and the reference method (upper arm oscillometry)

**Fig. 4:**
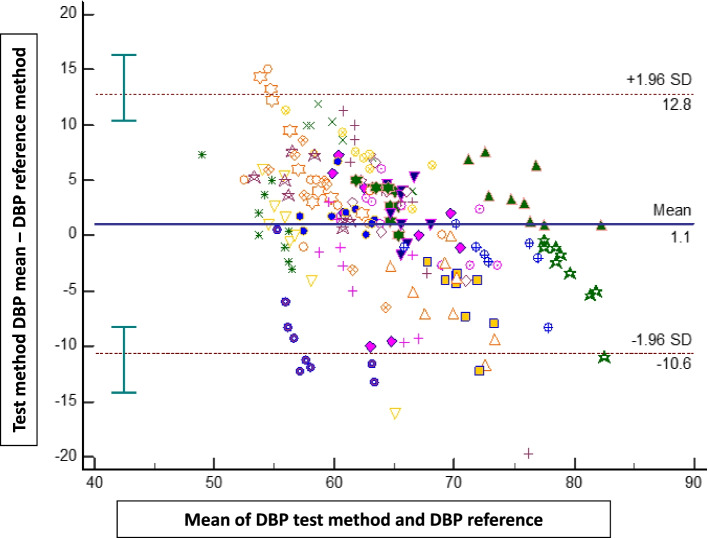
Bland–Altman plots showing the agreement between the smartphone application for diastolic blood pressure (DAP) measurements and the reference method (upper arm oscillometry)

The bias ± SD, 95% limits of agreement between both methods were 0.9 mmHg (± 7 mmHg; -13 to + 15 mmHg) for MAP, 0.2 mmHg (± 14 mmHg; -26 to + 27 mmHg) for SAP, and 1.1 mmHg (± 6 mmHg; -11 to + 13 mmHg) for DAP (Figs. 2, 3 and 4).

**Fig. 5:**
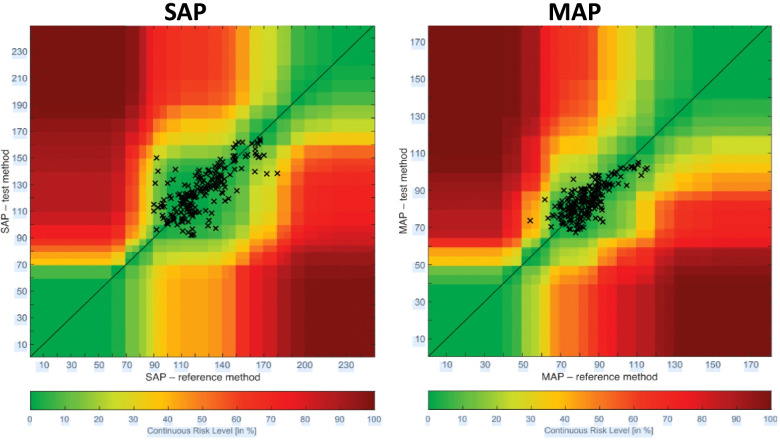
Error grid analysis comparing systolic (left panel) and mean (right panel) arterial blood pressure measurements from the smartphone application with those from the radial artery catheter (reference method). The background colors correspond to the continuous risk level for each pair of measurements. The continuous risk level ranges from 0 to 100% as shown at the bottom of the figure

The proportions of measurement pairs in risk zones A to E were 88.8% (no risk), 10% (low risk), 1% (moderate risk), 0% (significant risk), and 0% (dangerous risk) for MAP and 88.4%, 8.6%, 3%, 0%, 0%, respectively, for SAP (Fig. 5).

## Discussion

Our results indicate that there is relatively strong agreement between BP values collected using the OptiBP™ technology and those collected with an arterial catheter for patients in the ICU. When looking specifically at which comparisons met AAMI standards, MAP and DAP values were both successful while SAP did not meet criteria. Additionally, 99% of measurement comparisons were in either risk zone A (no risk) or B (low risk) for MAP and 97% were in the risk zones A or B for SAP.

There have been three previous studies investigating the OptiBP™ technology. The first study reported that the OptiBP™ measurements were accurate and precise compared to traditional double auscultatory oscillometric BP measurements in 50 patients measured in a hypertension department [17]. The mean of the differences was -1 ± 8 mmHg for SAP, -1 ± 5 mmHg for MAP and 0 ± 5 mmHg for DAP. Importantly, very strict research conditions were used with every patient sitting and placed in the same arm position. The next study was published by our group looking at emergency room patients and it demonstrated moderate accuracy between OptiBP™ and a standard upper arm cuff [18]. In a third study [19], conducted in patients admitted to a post-anesthesia care unit after intermediate risk surgery, there was good agreement between BP values obtained using the OptiBP™ and BP values obtained using an upper arm cuff. All these studies were performed using non-invasive reference methods that can be prone to some degree of imprecision. The current study used invasive arterial BP measurements as the reference, so additional accuracy for comparison is expected. Because of the study design (multiple time points over a two-day study period) using an invasive reference method, ICU patients were considered the most appropriate patient population, although the OptiBP™ application is not ultimately intended for ICU use.

The AAMI standards for non-invasive BP measurement state that, when comparing a new BP method with a reference method, a mean of the differences of less than 5 mmHg with a SD of ± 8 mmHg is clinically acceptable [21]. It is important to note that these standards were originally designed to assess non-automated, automated, and electronic sphygmomanometers, and not the technology used by the OptiBP™ system. Regardless, our results indicate that the OptiBP™ system met these criteria for MAP and DAP, while the SD of the SAP was unacceptably high.

Moving beyond AAIM criteria and the Bland–Altman analysis, we wanted to assess the clinical relevance of the findings in order to better facilitate potential future application. We decided that an error grid analysis with various clinical risk zones to indicate the likelihood of potential harm if the OptiBP™ values were used. At least 97% of every SAP and MAP value was located in the "no risk" or "low risk” zones, while ≤ 3% of measurements lay in the moderate risk zone (which is slightly higher than in our previous study but this is understandable as the population in the present study was more severely ill).

One notable strength of our current study is that multiple measurements at multiple times points were taken over a two-day study period in a population with expected variability in their blood pressure. However, the study also has weaknesses. Firstly, the initial calibration of the application requires a BP measurement with the reference method immediately before taking the measurements to be evaluated. As a result, all immediately subsequent BP values will most likely be quite similar to the recently recorded calibration measurement and any error in the method being tested would not be apparent. Secondly, the number of patients included in the study remains relatively small (30 patients) and the application provided measurements that could be analyzed in 73% of the included patients. It means that in 8 patients (27% of the study collective), no BP values could be measured. Reasons for inadequate signals are under investigation and may be multifactorial, such as artifacts related to poor positioning of smartphone on patient’s finger, inadequate signal due to bed / patient / smartphone movements, insufficient features recognition leading to signal non-processing by algorithm or morphological anomalies on finger of patient. However, this situation was anticipated given that the population tested was receiving intensive care, which likely effects peripheral vasculature. While not directly tested, judging the performance of OptiBP™ using results from ICU patients is not ideal as this population is often in poor general health, occasionally in the prone position, and potentially receiving vasoactive medications. This clinical setting is not what the algorithm was designed, trained or intended for, but our results still provide valuable information on the performance of the application because it is compared to an invasive gold standard in a population with higher BP variability than expected in the outpatient population. Thirdly, the portability (the ability of the application to be used on multiple smartphone models and operating systems) of the application needs to be addressed. Finally, among the patients included in this study, 64 had a hypertension during the study period, which means that 37% of patients did not have the condition for which this technology was developed. Moreover, as the sample size is relatively small, no subgroup analyses could be made (hypertension vs no hypertension or diabetes mellitus vs no diabetes mellitus).

In conclusion, in ICU patients, we observed a good agreement between BP values obtained using the OptiBP™ and BP values obtained using the radial artery catheter. OptiBP™ values fulfilled the exigency of the AAMI/ISO for MAP and DAP and error grid analysis revealed that the most measurement pairs for MAP (≥ 97%) were in risk zones A (no risk) and B (low risk). However, these promising results should be taken with caution as in 8 patients, no BP values could be measured and calibration was still necessary for the remaining 22. This smartphone application will therefore need some technical improvements moving forward before any clinical integration.

## Data Availability

All data of this study are not publicly available due to the development of this new smartphone application but can be obtained by request to the corresponding author.

## Abbreviations

MAP: Mean arterial pressure
ICU: Intensive care unit
BP: Blood pressure
OptiBP: Optical blood pressure
PPG: Photoplethysmography
AAMI: Association for the advancement of medical instrumentation
